# Endothelial Cells Potentiate Interferon-γ Production in a Novel Tripartite Culture Model of Human Cerebral Malaria

**DOI:** 10.1371/journal.pone.0069521

**Published:** 2013-07-12

**Authors:** Loke Tim Khaw, Helen J. Ball, Jacob Golenser, Valery Combes, Georges E. Grau, Julie Wheway, Andrew J. Mitchell, Nicholas H. Hunt

**Affiliations:** 1 School of Medical Sciences and Bosch Institute, University of Sydney, Sydney, Australia; 2 Department of Microbiology and Molecular Genetics, The Kuvin Center for the Research of Tropical and Infectious Diseases, The Hebrew University of Jerusalem, Jerusalem, Israel; 3 Department of Parasitology, Faculty of Medicine, University of Malaya, Kuala Lumpur, Malaysia; Université Pierre et Marie Curie, France

## Abstract

We have established a novel *in vitro* co-culture system of human brain endothelial cells (HBEC), *Plasmodium falciparum* parasitised red blood cells (iRBC) and peripheral blood mononuclear cells (PBMC), in order to simulate the chief pathophysiological lesion in cerebral malaria (CM). This approach has revealed a previously unsuspected pro-inflammatory role of the endothelial cell through potentiating the production of interferon (IFN)-γ by PBMC and concurrent reduction of interleukin (IL)-10. The IFN-γ increased the expression of CXCL10 and intercellular adhesion molecule (ICAM)-1, both of which have been shown to be crucial in the pathogenesis of CM. There was a shift in the ratio of IL-10:IFN-γ protein from >1 to <1 in the presence of HBEC, associated with the pro-inflammatory process in this model. For this to occur, a direct contact between PBMC and HBEC, but not PBMC and iRBC, was necessary. These results support HBEC playing an active role in the pathogenesis of CM. Thus, if these findings reflect the pathogenesis of CM, inhibition of HBEC and PBMC interactions might reduce the occurrence, or improve the prognosis, of the condition.

## Introduction

Malaria continues to be one of the most significant infectious diseases in the world, assailing developing countries in terms of both morbidity and mortality. Cerebral malaria (CM) is the most severe manifestation of *Plasmodium falciparum* malaria infection with an average mortality rate of around 20% even when treated with anti-malarial drugs [1], [2]. Despite decades of study, a detailed understanding of the causative mechanisms in CM has so far not been achieved. Studies of CM can be categorised into four broad types [3]: clinical or genetic studies undertaken in malaria endemic areas, *in vivo* experiments utilising animal models, histopathological studies on post-mortem materials and *in vitro* investigations of the interactions between the cell types that contribute to the disease.

Clinical studies have often involved measuring cytokines or other biomarkers in the serum/plasma [4], [5], [6] and cerebrospinal fluid (CSF) from malaria patients [7]. They also include the study of post-mortem material (brains) from patients who succumbed to the disease. Another aspect of clinical work is investigation of the neurological sequelae in survivors of CM. Experimental *in vivo* studies, on the other hand, involve the use of animal models to study CM. Even though differences between human and murine CM have been described [8], [9], the animal model has proven to be versatile and revealing, in particular with gene ablation studies, where inferences can be made by comparing gene knockout mice to wild type mice in their response towards the disease. An important finding originating from this approach is that the pro-inflammatory cytokine interferon-γ (IFN-γ) is crucial for the pathogenesis of experimental CM [10], [11], [12].

*In vitro* cultures also have been performed, utilising selected cells observed in the CM lesion, such as brain endothelial cells, peripheral blood mononuclear cells, platelets and parasitised red blood cells [13]. This allows the study of interactions between different cell types. These studies largely have been limited to bipartite cultures, which do not fully represent the cellular components of the CM lesion. Some studies that have used human brain endothelial cells, platelets and iRBCs *in vitro* have revealed roles for platelets in the pathogenesis of CM in tripartite cultures [14], [15], [16], [17], [18]. However, PBMCs have yet to be included in a tripartite culture system to model the lesion in CM.

Hence, for this study, we established a novel tripartite culture, using human PBMCs, iRBCs and HBEC, in order to simulate the vascular lesion of CM. We hypothesised that PBMCs, along with HBEC, would interact with the iRBCs, leading to up-regulation of the expression of inflammatory genes.

## Results

### 1. Endothelial cells (HBEC-5i) enhance IFN-γ production, but decrease that of IL-10, in PBMC/ 3D7 iRBC co-cultures

**Figure 1:**
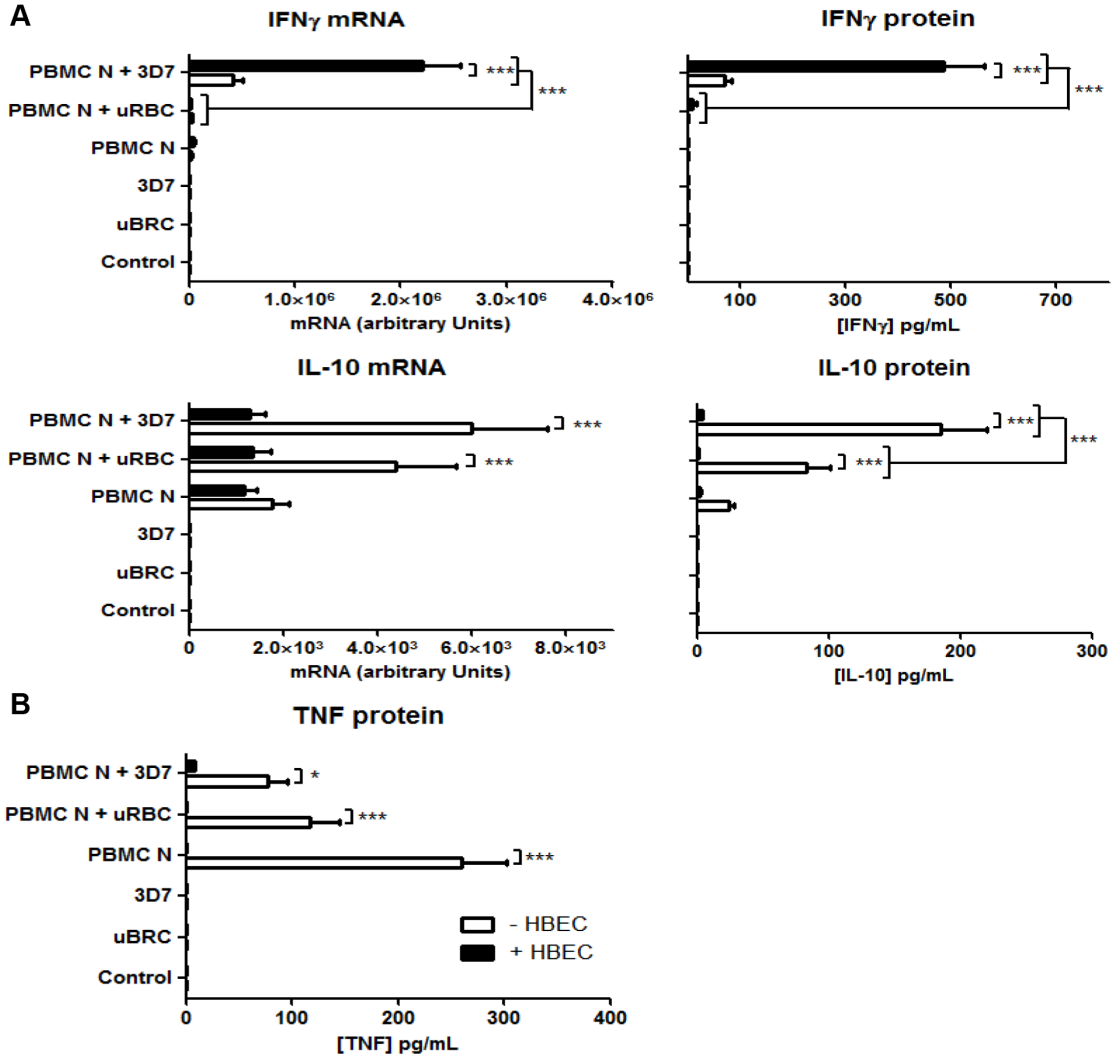
Effect of endothelial cells on cytokine production in PBMC/iRBC co-cultures. A. Significant alterations in IFN-γ and IL-10 (mRNA and protein) production in PBMC/iRBC (*P. falciparum* strain 3D7) co-culture in the presence of endothelial cells (HBEC-5i). B. TNF protein production was significantly decreased in the presence of HBEC-5i. uRBC =  unparasitised red blood cells. Control  =  no PBMC, uRBC or iRBC. Columns and horizontal bars represent means ± SEM of nine experiments. Two-way ANOVA showed significant differences among the groups (*p<0.05; ***p<0.001), identified using the Bonferroni post hoc test.

In nine separate experiments with the novel tripartite cultures of HBEC, PBMCs (from donor N) and iRBC (strain 3D7), IFN-γ mRNA expression was significantly enhanced when endothelial cells were present (PBMC N + 3D7 + HBEC, Figure 1A). IFN-γ protein expression echoed that of mRNA, with a 6.8-fold enhancement in cultures with HBEC-5i compared to PBMC + iRBC without endothelial cells (Figure 1A). This effect was parasite-dependent, since significant increases of IFN-γ mRNA and protein were not observed in the corresponding controls of HBEC + PBMC, PBMC only, HBEC + PBMC + uRBC (uRBC =  uninfected red blood cells) and PBMC + uRBC. The results suggest that HBEC amplified the induction of IFN-γ expression by PBMC in this co-culture arrangement.

The expression of an anti-inflammatory cytokine, IL-10, in the tripartite culture system was reduced. Production of this cytokine, in terms of protein (Figure 1A), but not mRNA, was dependent on the presence of parasitised red blood cells. Both IL-10 mRNA and protein, however, were significantly suppressed in the presence of endothelial cells, again implying that HBEC exert an overall pro-inflammatory effect in this system.

Expression of the cytokine TNF, like IL-10, was significantly reduced in the presence of HBEC (Figure 1B). This was unexpected, given that TNF expression can be regulated by IFN-γ.

### 2. Heterogeneity between PBMC donors, but not parasite strains, in IFNγ production in co-culture

**Figure 2:**
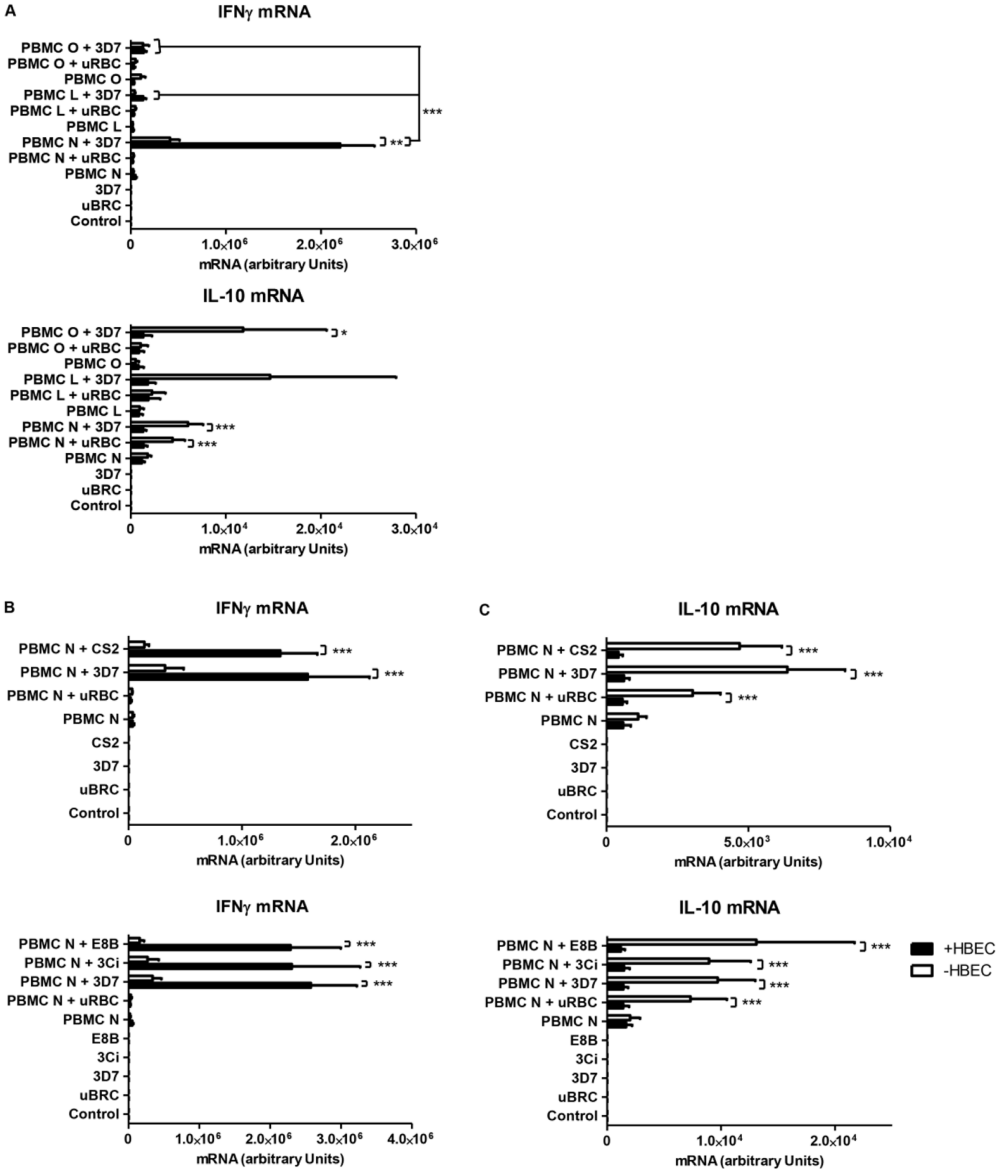
Influence of PBMC origin and malaria parasite strain on cytokine production in tripartite cultures. A. Differences in the pattern of IFN-γ, but not IL-10, mRNA production between donor PBMCs (L, N and O) in tripartite culture. B: No difference in the pattern of IFN-γ expression in response to *P. falciparum* strains 3D7, CS2, 3Ci and E8B in the tripartite culture. C: Similar pattern of IL-10 expression with several *P. falciparum* strains in the tripartite culture. uRBC  =  unparasitised red blood cells. Control  =  no PBMC, uRBC or iRBC. Columns and horizontal bars represent means ± SEM of three to nine experiments. Two-way ANOVA showed significant differences among the groups (*p<0.05; **p<0.01; ***p<0.001), using the Bonferroni post hoc test.

Following the assessment of the cytokine responses of the initial donor PBMC (designated “PBMC N”), two further PBMC donors (designated PBMC L and O) were tested for their capacity to induce IFN-γ in the tripartite cultures. Heterogeneity in the IFN-γ response between donors in bipartite incubations of PBMC and iRBC has been reported by others [19], [20], [21], [22]. PBMC L and O were selected from among 6 donors for their low, or lack of, reactivity towards 3D7 iRBC in a preliminary experiment in terms of IFN-γ mRNA (data not shown). As hypothesised, heterogeneity in the IFN-γ response was seen, with PBMC L and PBMC O showing initial lower induction of IFN-γ mRNA in the tripartite culture compared to PBMC N. In contrast, there was no difference in the pattern of IL-10 mRNA expression between the three donors (Figure 2A).

Different laboratory maintained strains of *P. falciparum* (3D7, CS2, 3Ci and E8B) were then tested for their ability to elicit IFN-γ and IL-10 production in the tripartite culture system. No heterogeneity between the strains was observed in the context of the tripartite culture (Figures 2B and 2C), as the patterns of expression of IFN-γ and IL-10 mRNA were similar to those in Figure 1A.

### 3. ICAM-1 and CXCL10 expression is IFN-γ dependent

To demonstrate the functional capacity of the IFN-γ produced by the tripartite culture, the expression of two of its downstream effectors, ICAM-1 and CXCL10, was studied. Both of these are considered to be important in the pathogenesis of CM. ICAM-1 is acting as a major adhesion molecule for iRBC [23], [24] as well as leukocytes, possibly via LFA-1 [25], [26]. CXCL10 functions as a chemokine and deletion of its receptor, CXCR3, is protective in experimental cerebral malaria [27]. We employed an antibody that neutralises IFN-γ in order to determine whether production of these downstream effectors was regulated by IFN-γ in the tripartite culture.

**Figure 3:**
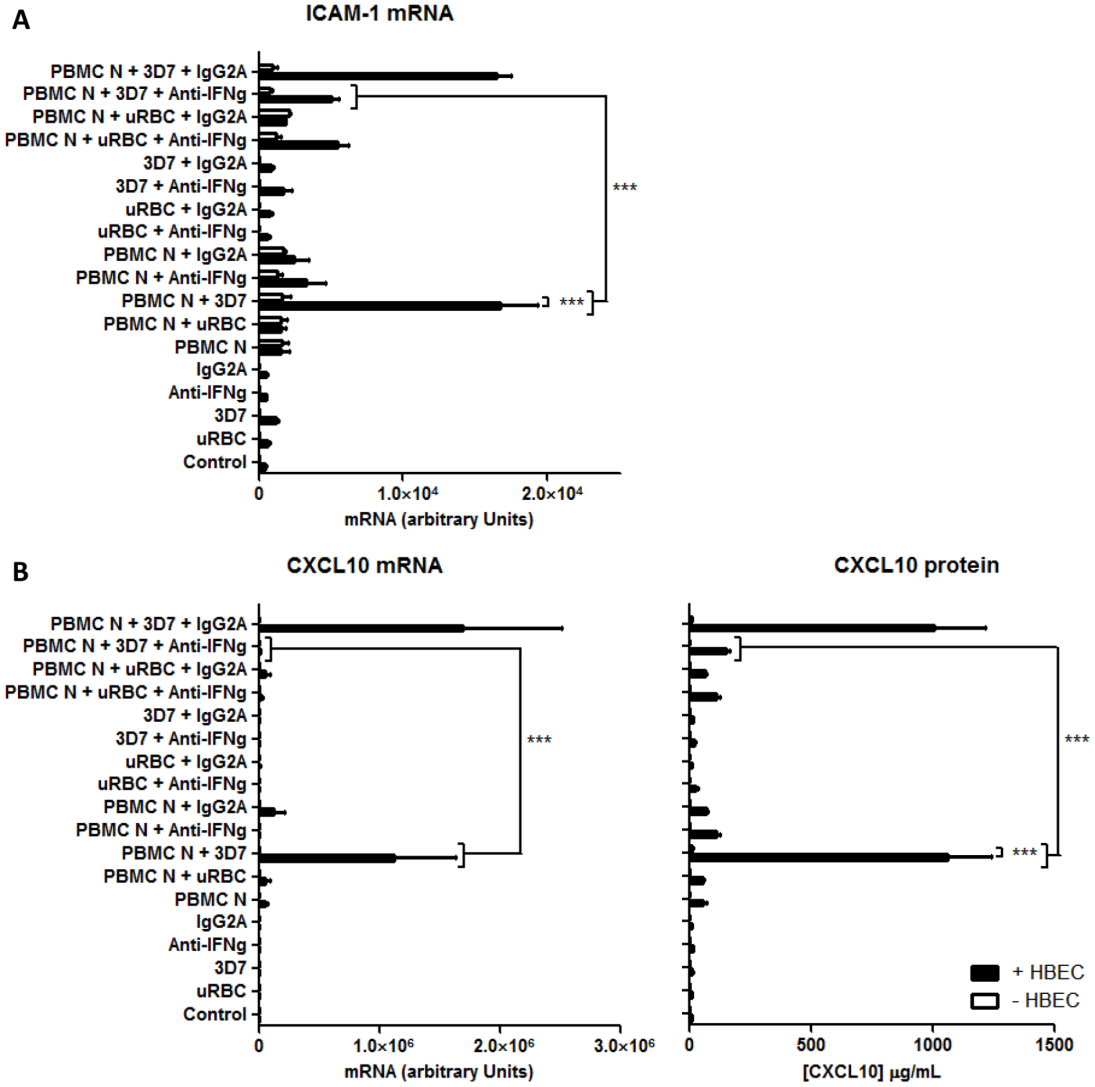
HBEC-dependent induction of ICAM-1 and CXCL10 in tripartite co-cultures is inhibited by IFN-γ neutralising antibody. A. IFN-γ neutralising antibody inhibits ICAM-1 mRNA induction in tripartite cultures. B. IFN-γ neutralising antibody inhibits expression of CXCL10 mRNA and protein in the tripartite cultures. uRBC  =  unparasitised red blood cells. Control  =  no PBMC, uRBC or iRBC. Columns and horizontal bars represent means ± SEM of three experiments. Two-way ANOVA showed that the values contained significant differences among the groups (***p<0.001), using ANOVA and the Bonferroni post hoc test.

As predicted, ICAM-1 mRNA induction was enhanced in the tripartite arrangement. This mRNA expression was reduced by 70% following the addition of IFN-γ neutralising antibody (Figure 3A), suggesting that the ICAM-1 induction is IFN-γ dependent. CXCL10 expression in PBMC induced by 3D7 iRBCs was potentiated in the presence of HBEC, with 500- and 91-fold increases of mRNA and protein respectively compared to observations in the absence of endothelial cells (Figure 3B). The addition of IFN-γ neutralising antibody abrogated CXCL10 expression. In contrast, addition of isotype-matched control antibodies did not affect ICAM-1 or CXCL10 production (Figure 3A, B).

### 4. IL-10:pro-inflammatory cytokine ratios are decreased in the presence of HBEC

**Figure 4:**
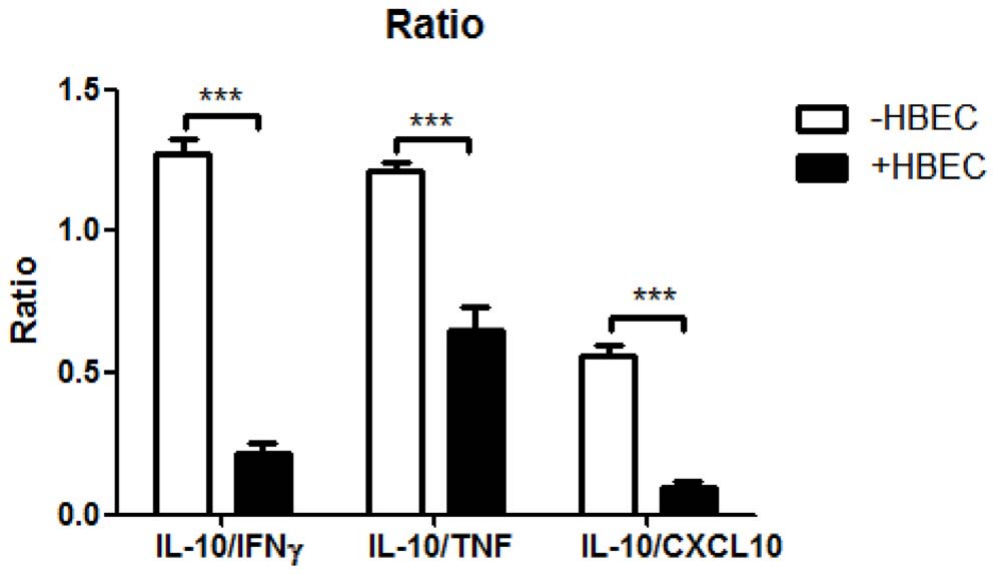
Ratio of IL-10:IFN-γ, IL-10:TNF and IL-10:CXCL10 protein in the presence and absence of HBEC. Columns and vertical bars represent means ± SEM of three experiments. Statistical significance (***p<0.001) was assessed using ANOVA and the Bonferroni post hoc test.

It has been suggested that a reliable predictor of malaria severity is comparison of the ratio between TNF and IL-10 protein levels in the plasma [28], [29]. A ratio of IL-10:TNF of less than 1 correlated with severe malaria whereas a ratio of more than 1 was associated with uncomplicated malaria [29]. In our study, in the culture supernates with HBEC, we observed significant decreases, from >1 to <1, in the ratio of IL-10:TNF as well as IL-10:IFN-γ proteins (Figure 4). In addition, although the ratio in the absence of HBEC is already lower than 1, we found a similar significant reduction of the IL-10: CXCL10 ratio when HBEC were added into the system.

### 5. IFN-γ enhancement is caspase-1 independent

**Figure 5:**
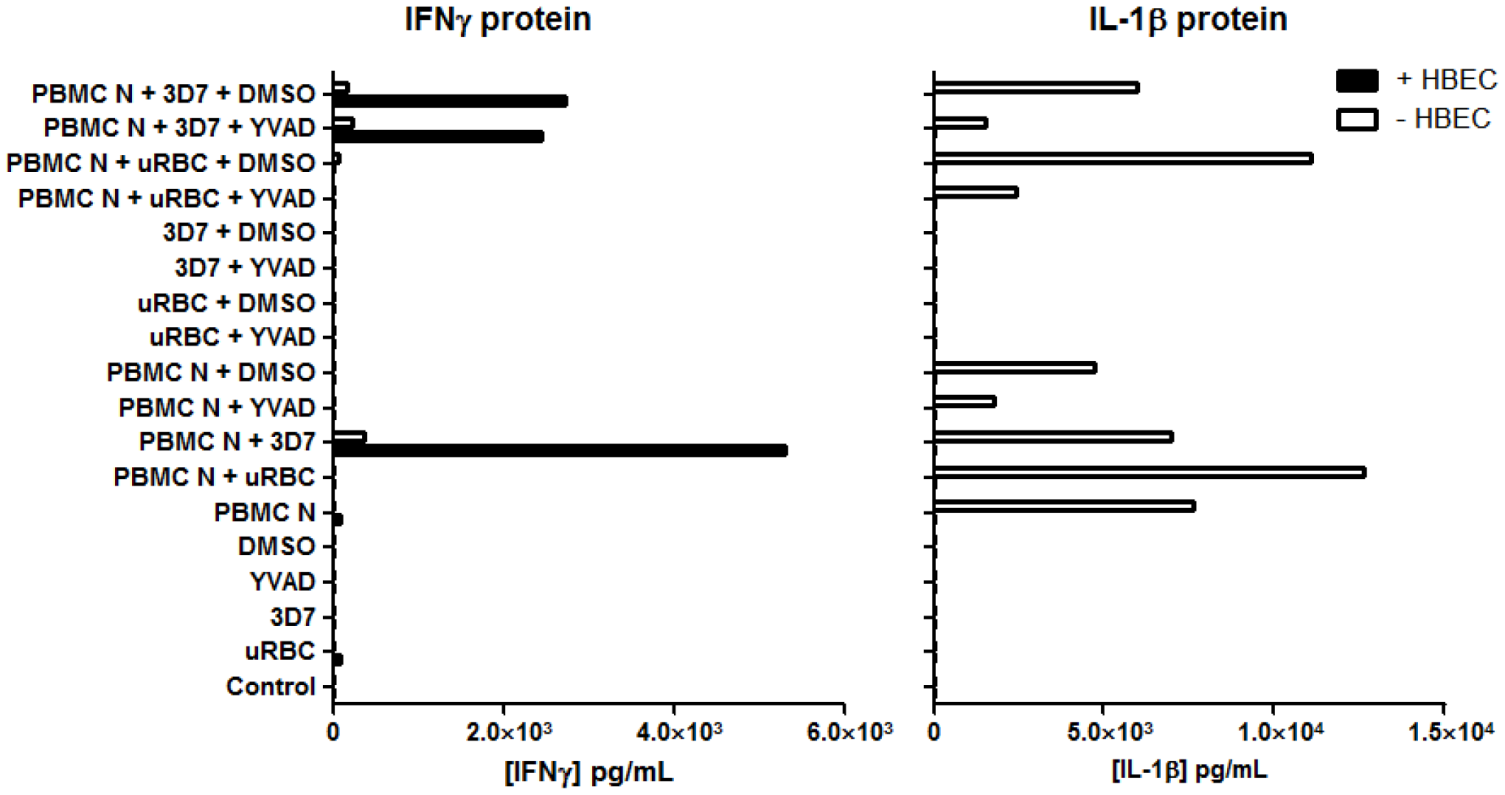
Effect of caspase-1 inhibition on IFN-γ and IL-1β production in co-cultures of PBMC and iRBC. YVAD did not affect IFN-γ production but did inhibit that of IL-1β. uRBC = unparasitised red blood cells. Control  =  no PBMC, uRBC or iRBC. YVAD (50 µmol/L), a caspase-1 inhibitor, was pre-incubated with PBMC and HBEC for 30 min prior to addition of the iRBCs. At 24 h, supernates were analysed for IFN-γ and IL-1β. Columns represent values from a single experiment.

To study the upstream events leading to the expression of IFN-γ in this co-culture system, the potential involvement of cytokines such as IL-1β, IL-12 and IL-18 was explored. All these cytokines provide co-stimulatory signals that elicit the expression of IFN-γ from immune cells, including NK cells [30]. To determine whether IL-18 and IL-1β are crucial for IFN-γ expression in this tripartite culture model, YVAD, an inhibitor of caspase-1, was used to inhibit caspase-1-mediated activation of pro-IL-1β and pro-IL-18 to their active forms [31]. YVAD did not affect IFN-γ protein level in the tripartite cultures (PBMC + HBEC + iRBC +YVAD) (Figure 5) when compared to its DMSO control (PBMC + HBEC + iRBC + DMSO). In addition to IFN-γ, IL-1β production was analysed to verify the activity of YVAD in this system. Addition of YVAD did indeed reduce the level of IL-1β in culture supernates (Figure 5). These results imply that IFN-γ production is independent of caspase-1 activity and, hence, IL-18 in this co-culture system. We were not able to quantify IL-18, due to technical limitations of the FlowCytomix multiple analyte detection system employed. IL-12 on the other hand, was undetectable in the supernates in our model, and hence was not pursued further (data not shown).

### 6. Enhancement of IFN-γ production by HBEC is contact dependent

**Figure 6:**
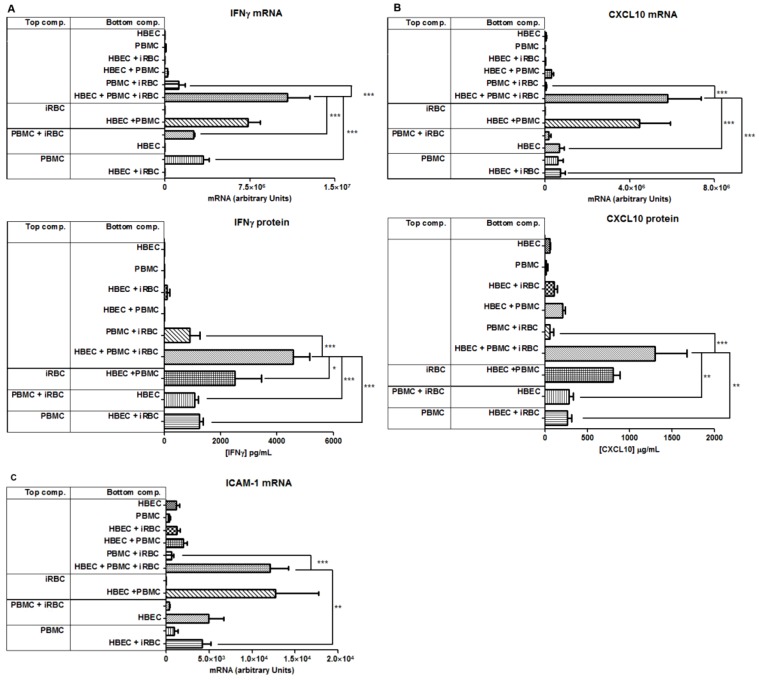
Effect on immunomodulator expression of the physical separation of cellular components of the tripartite system. The enhancement of IFN-γ (A), CXCL10 (B) and ICAM-1 (C) production was perturbed when PBMC were physically separated from HBEC. iRBCs, iRBCs + PBMCs or PBMCs were isolated in the upper chamber of the transwell insert, whereas HBEC were consistently retained in the bottom chamber. Columns and horizontal bars represent means ± SEM of three experiments. One way ANOVA showed significant differences among the groups (*p<0.05; **p<0.01; ***p<0.001), using Tukey's post hoc test.

As we did not find evidence for IFN-γ expression being cytokine mediated, we examined the contact dependency between the different cell types in the induction of IFN-γ in the tripartite culture, using transwell inserts (pore size of 0.4 µm). HBEC were always situated in the lower chamber. Preventing physical contact of PBMCs with other cellular components, by isolating them in the upper chamber, reduced the amount of IFN-γ mRNA by 70%, compared to control (p<0.001, Fig. 6A). This was also observed when both iRBCs and PBMCs were separated in the upper chamber (Figure 6A). The separation of the iRBCs in the upper chamber, and co-culture of PBMC and HBEC in the lower chamber, did not significantly reduce IFN-γ mRNA expression. These data imply that *P. falciparum* stimulates IFN-γ production in the tripartite culture in a contact independent fashion, likely through released antigens or other soluble factors. This same pattern of expression was observed at the protein level (Figure 6A), with one exception, as separation of the iRBC from the HBEC and PBMC resulted in a modest but significant reduction in IFN-γ expression, by about 40% (Figure 6A). These results indicate that PBMCs are required to be in close contact with HBEC in order to evoke maximal amounts of IFN-γ. Importantly, the data confirm that PBMCs, rather than HBEC, are the source of IFN-γ in this system.

Similar to the effects on IFN-γ, isolation of PBMC from the other culture components considerably reduced both CXCL10 and ICAM-1 levels. If the upper chamber contained either PBMC alone, or PBMC and iRBC together, the amount of CXCL10 mRNA and protein was reduced (Figure 6B), suggesting that contact between PBMC and HBEC is essential to generate the maximal amounts of CXCL10 in the presence of iRBCs. These data also suggest that the source of CXCL10 mRNA may be both PBMC and HBEC, as both cell types expressed similar levels even when separated (Figure 6B). In parallel to the patterns of expression seen with CXCL10, ICAM-1 mRNA was significantly diminished by 70% when PBMC were separated in the upper chamber compared to when all components were cultured together in the lower chamber (Figure 6C). Again, isolation of iRBCs in the upper chamber had no effect on ICAM-1 mRNA production. Culture of both iRBCs and PBMCs together in the upper chamber also resulted in an apparent, but not significant, reduction of ICAM-1 mRNA. Finally, the induction of ICAM-1 mRNA was shown to be occurring mainly in HBEC, rather than PBMCs, as demonstrated in Figure 6C when HBEC were separated from PBMCs by the transwell inserts. Overall, these data support a model where HBEC and PBMCs require close contact in order to potentiate IFN-γ, and subsequently CXCL10 and ICAM-1, expression.

### 7. IFN-γ enhancement is substantially dependent on NK cells

**Figure 7:**
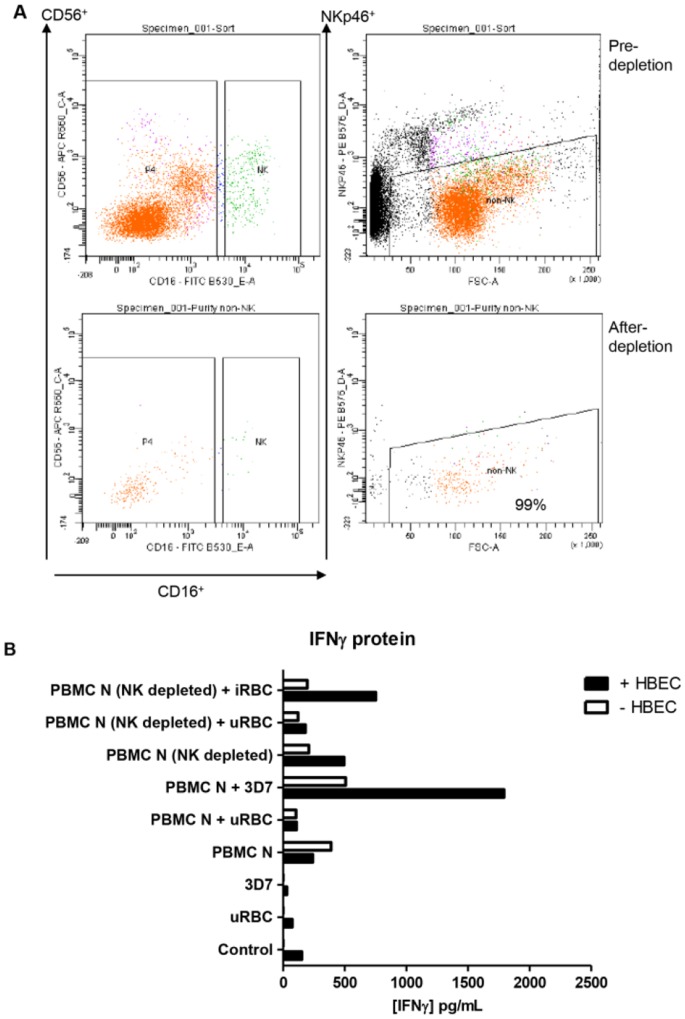
Effect of NK depletion on IFN-γ production in tripartite cultures. A. NK cell depletion strategy. B. IFN-γ protein production was decreased by NK cell depletion. uRBC  =  unparasitised red blood cells. Control  =  no PBMC, uRBC or iRBC. “PBMC N (NK depleted)” co-cultures contained PBMC that had been depleted of 99% NK cells stained with CD16, CD56 and NKp46 prior. “PBMC N” refers to co-cultures in which PBMC had not been depleted of NK cells. Co-cultures were 24 h, following which supernates were harvested and analysed for IFNγ. Columns represent means of two separate experiments.

To narrow down the possible ligands and receptors responsible for the phenomenon of endothelial cell enhancement of IFN-γ production, we used a cell-sorting approach to deplete selected subsets in the PBMC stabilates prior to our standard co-culture experiments. As the literature has described a range of cellular sources (T, NK, NKT and γδ T cells) as being responsible for IFN-γ production in the response of PBMC to parasitised red blood cells in bipartite cultures [32], [33], [34], an ideal approach would have involved depletion of all subsets in turn. However, due to limited stocks of the relevant PBMC, we focused on NK cells, as these have been the most widely-reported source. Using the NK specific markers CD16^+^, CD56^+^ and NKp46^+^, and the gating strategy shown in Figure 7A, PBMC were depleted of NK cells (>99% purity) and subsequently cultured with iRBC and HBEC. In two independent experiments, IFN-γ protein production was greatly reduced in NK-depleted PBMC cultures compared to the corresponding co-culture from which NK cells were not depleted, PBMC N + 3D7 + HBEC (Figure 7B). These data indicate that NK cells were involved in the production of IFN-γ in the tripartite culture.

### 8. IFN-γ production is co-stimulated by human brain endothelial cells, potentially via MHCII, ICOSL, IL-2 and B7-1/B7-2

**Figure 8:**
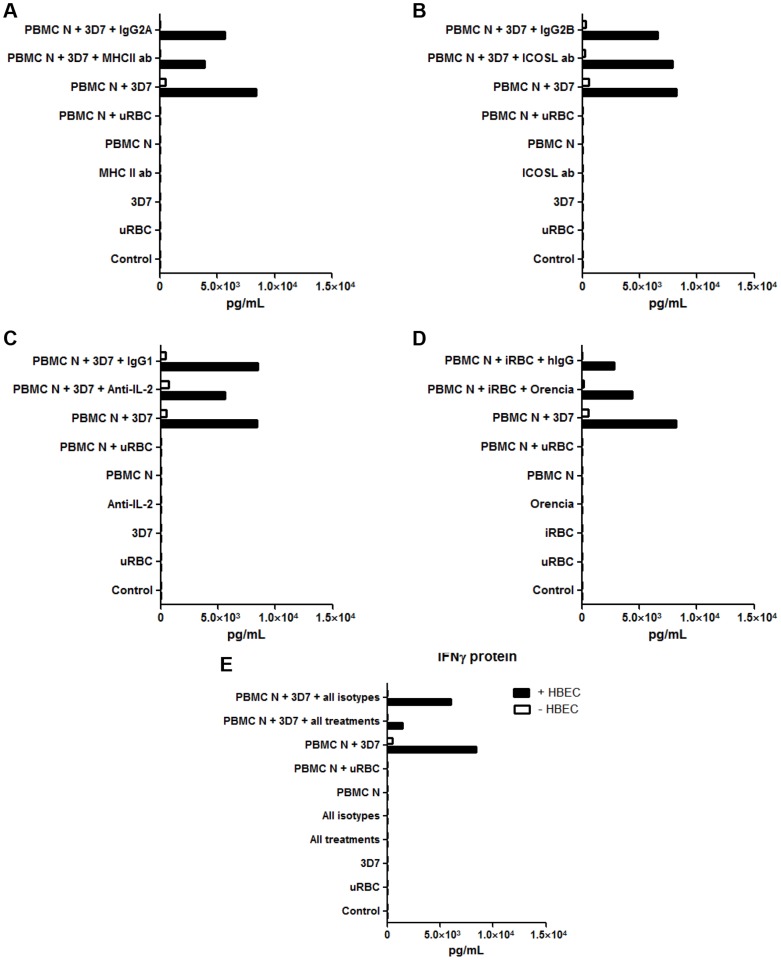
Effects of MHC II, ICOSL, IL-2 neutralisation on IFN-γ production in the tripartite cultures. Individually, anti-MHC II (A), anti-ICOSL (B), anti-IL-2 (C) and Orencia (D) did not affect IFN-γ production. However, when all treatments were combined they did reduce the endothelial cell-mediated enhancement of IFN-γ (E). uRBC  =  unparasitised red blood cells. Control  =  no PBMC, uRBC or iRBC. MHC II, ICOSL and IL-2 neutralising antibodies, and Orencia, were incubated with the tripartite cultures for 24 h. Supernates were then harvested and analysed for IFN-γ. Columns represent means of two separate experiments.

Having shown that NK cells were partly responsible for the increase of IFN-γ protein in the tripartite culture, we next explored the possible involvement of several molecules in this system. Those studied were the major histocompatibility complex II (MHC II), Inducible costimulator molecule ligand (ICOSL), interleukin-2 (IL-2) and B7-1/B7-2. The first three were tested by the addition to co-cultures of neutralising antibodies. B7-1/B7-2 actions were blocked with Orencia (Abatacept), a fusion protein of the Fc region of IgG1 and the extracellular domain of CTLA-4. Pooled data from two independent experiments showed that MHCII, ICOSL, IL-2 and B7-1/B7-2 inhibition individually did not affect IFNγ expression in tripartite cultures, compared to their respective isotype controls (Figure 8A-D). However, when administered together, the treatments decreased IFN-γ expression to almost control levels in two separate experiments (Figure 8E), suggesting that two or more of the four molecules may act in concert to stimulate IFN-γ expression.

## Discussion

The mechanisms leading to the pathology seen in CM are still the topic of considerable debate. This study presents, for the first time, a novel tripartite culture system consisting of three major cell types involved in the lesion of CM: brain endothelial cells, iRBC and PBMC. We wished to determine whether endothelial cells would modulate the production of immune mediators by PBMC stimulated by iRBC. This might throw light on the interactions of these three cell types at a critical location, the brain microvasculature. The primary focus in this model was on the local expression of IFN-γ, a pro-inflammatory cytokine that is generally accepted to be crucial in driving the pathogenesis of CM [13], [35], [36], [37] as well as being central to anti-malarial immunity [38]. Clinical studies have described a correlation between IFN-γ levels and the occurrence of CM in humans [39], [40], [41]. Based on animal studies, this IFN-γ appears to derive from immune cells that have accumulated within the brain vasculature prior to the terminal phases of pathology. Previously, innate production of IFN-γ by PBMCs in response to iRBC has been reported by a number of groups, although this has been limited to bipartite cultures consisting of PBMC and iRBC. More importantly, design and interpretation of these studies has focused primarily on understanding the cellular and molecular pathways involved in IFN-γ production, rather than any effects on pathology. Taken together these studies suggest that production of IFN-γ by leukocytes sequestered within brain microvessels, and therefore in close contact with endothelial cells, is important for CM pathology.

Since suitable human experimental systems for investigating the role of IFN-γ in the pathogenesis of CM are not available, such investigations are limited to animal models. In animal studies using *P. berghei* ANKA, IFN-γ has been shown to be critical for the development of experimental CM (ECM). Neutralisation of IFN-γ in mice infected with *P. berghei* ANKA protects against the manifestation of ECM [42], and mice that are deficient in IFN-γ were also resistant to ECM [12], [43], [44]. The mechanisms by which IFN-γ drives pathology in this model are likely to be multiple. Firstly, tissue sequestration of parasites, including to the brain, is mediated by IFN-γ [45]. More relevant to the current study, IFN-γ-induced chemokines, in particular CXCL10, are crucial for the migration of leukocytes to the brain [11], [27].

Here, in the tripartite culture model, our results showed a novel role of endothelial cells in potentiating IFN-γ production by PBMC in response to iRBC, and subsequent IFNγ-dependent processes that have been implicated in CM. It should be noted that we studied only a 24 hour co-incubation period, and it is possible that the results would have been different at other time points. An important finding of our co-culture system is that expression of both CXCL10 and ICAM-1 mRNA was enhanced in the presence of endothelial cells in an IFN-γ- and contact-dependent manner. CXCL10, also known as IP-10 (Interferon gamma induced protein 10), signals through CXCR3 and is a key chemokine involved in T helper and T cytotoxic cell migration [46], [47]. The relevance of CXCL10 to the pathogenesis of CM is highlighted by studies showing that CXCL10 is markedly increased in the plasma of fatal CM cases as compared to other non-CM cases [48]. Furthermore, mice deficient in CXCL10 or CXCR3 are partially resistant to CM [11], [27], [49], [50]. ICAM-1 is an adhesion molecule that has been implicated in the development of murine CM. ICAM-1 is a host receptor for the *Pf*EMP1 molecule of iRBCs [51], so an increase in ICAM-1 expression is likely to lead to sequestration of iRBCs as well as the recruitment of more PBMCs, potentially leading to CM. This was supported recently by human studies in which ICAM-1 binding by iRBCs was associated with CM [23]. Although TNF has been considered the primary inducer of ICAM-1 in CM [52], [53], there are also reports that suggest that IFNγ provides a stronger signal for induction of this molecule *in vitro* [54], [55]. Importantly, in our study neutralisation of IFN-γ reduced expression of ICAM-1 and CXCL10 mRNA to almost control levels (Figure 3), which further supports a role for IFN-γ in the pathogenesis of CM.

The results shown here were obtained with PBMC from a naive donor that reacted strongly to iRBC in terms of IFN-γ induction. Such heterogeneity in IFN-γ responsiveness is not surprising, considering that only a very small fraction of *P. falciparum*-infected patients develop CM. Indeed, it is well-established that PBMC from malaria-naïve donors show a very wide range of responsiveness to iRBC in terms of IFN-γ production [41], [56], [57]. Furthermore, since a range of *P. falciparum* strains induced similar levels of IFN-γ in tripartite co-culture, it is likely that the strains used in this study may share a common factor that is crucial for the induction of IFN-γ and that host, rather than parasite, factors may be the dominant contributors to CM pathology.

In contrast to the clearly pro-pathogenic role of IFN-γ in CM, the influence of IL-10 and TNF on disease is unclear, but IL-10 levels in particular have been argued to be a prognostic indicator of protection from pathology. The actual role of IL-10 is debatable, as comparisons of plasma samples from children afflicted with mild and severe forms of malaria showed that IL-10 levels were significantly lower in patients with the more severe forms of malaria [58], [59], though it must be noted that another study did not support this finding [60]. Any protective effect of IL-10 is presumably a result of the anti-inflammatory activity of this cytokine [61]. TNF was initially deemed important in CM [4], [62], but abrogation of the TNF gene in the murine model of CM did not confer resistance to CM [63]. Nevertheless, the IL-10:TNF ratio in patient plasma has been shown to correlate predictively with severe disease [29]. In our tripartite co-culture system, inclusion of endothelial cells lead to diminished IL-10 production, which when coupled with the enhanced IFN-γ production, led to a dramatic inversion in the IL-10:IFN-γ ratio in the presence of HBEC. Similar, but less pronounced, changes in the IL-10:TNF and IL-10:CXCL10 ratios were also seen in our culture system, which again is consistent with a pro-pathogenic function of endothelial cells.

The pro-inflammatory activity of the endothelial cells was mediated through cell-cell contact rather than soluble mediators. When PBMCs were separated from the tripartite cultures in transwells (Figure 6A, 6B and 6C), IFN-γ mRNA and protein, as well as CXCL10 and ICAM mRNA levels, were reduced. Taken together, these data indicate that in the tripartite cultures, up regulation of IFN-γ, ICAM-1 and CXCL10 induction requires contact-dependent signals between PBMC and HBEC. Interestingly, the separation of the iRBCs from other culture components did not significantly reduce the amount of IFN-γ mRNA, suggesting that neither cytoadherence of iRBC to HBEC nor direct effects of released merozoites are important in this mechanism.

The source of IFN-γ in *P. falciparum* co-culture systems has been a matter of intense debate, with NK cells [21], [22], [26], [57], [64], [65], CD8^+^ T cells [34] and γδ T cells/ NKT cells [33] being argued to be the dominant source. Of the likely parasite factors involved in immune stimulation, it has been shown that glycosylphosphatidylinositol [66], hemozoins [67], [68], [69], uric acid [70] as well as microparticles [71] are capable of eliciting pro-inflammatory activity such as expression of IFN-γ and TNF. To determine the source of IFN-γ in our studies, we focused on NK cells as the majority of published studies support them being the major producers of this cytokine. NK depletion experiments (Figure 7A and 7B) showed that NK-depleted PBMC produced lower levels of IFN-γ protein in the tripartite culture system, when compared to NK replete PMBC cultures. Nevertheless, PBMCs that had been depleted of NK cells still produced residual amounts of IFN-γ even in the absence of other culture components. One possible explanation for this apparent heterogeneity of cellular source is that IFN-γ production by various cell types may be time-dependent, with 24 hours culture (the time point used in the present study) marking a transition from NK to CD4 T cells as the dominant source [34].

As far as interactions between endothelial cells and NK cells are concerned, potential co-stimulatory molecules on endothelial cells have been extensively reviewed [72] and these include ICOSL (inducible co-stimulator ligand) and PD-1 L (programmed death 1 ligand). On the other hand, NK cells possess a variety of receptors such as CD28, a ligand of B7-1/B7-2 molecules [73], which is expressed by endothelial cells as well as monocytes and dendritic cells. Another molecule of interest is MHC II, which, apart from being involved in antigen presentation, has also been described as being capable of synergising with Toll-like receptors in mounting innate immune responses [81], [82]. Furthermore, higher inducibility of MHC II expression in murine brain endothelial cells is associated with genetic susceptibility to CM in one model of ECM [74]. Even though we did not find any significant reduction in IFN-γ protein in the tripartite cultures with single additions of MHC II, ICOSL, IL-2 neutralising antibodies and Orencia, a B7-1/B7-2 antagonist, when these were added together IFN-γ induction was largely abolished (Figure 8E). This evidenced the potential of these molecules to act in concert for the induction of IFN-γ by HBEC in the presence of both iRBCs and PBMC. Our findings so far suggest that endothelial cells provide co-stimulatory signals in this co-culture system.

## Conclusion

We describe for the first time a novel tripartite culture using HBEC, PBMC and iRBC. In this co-culture system, HBEC actively participate in the pro-inflammatory response by enhancing IFN-γ production, which in turn leads to induction of a chemokine (CXCL10) and a cell surface receptor (ICAM-1) that have been implicated in CM pathology. These results indicate a need to further investigate the role of endothelial cells in end stage CM pathology, and suggest that therapeutic interventions that target endothelial cell/leukocyte interactions may be possible.

## Materials and Methods

### Ethics statement

Human buffy coat preparations and outdated erythrocytes were purchased from the Red Cross Blood Service, Sydney. Donors gave written informed consent. The studies were approved by the University of Sydney Human Ethics Committee.

### P. falciparum culture

*P. falciparum* cultures were maintained using methods modified from those described by Trager and Jensen [75]. In brief, parasites were grown in group O^+^ human erythrocytes in Malaria Complete Medium (MCM) consisting of RPMI 1640 fortified with 2 mmol/L glutamine (Thermo Fisher Scientific, USA), glucose (10 mmol/L, Amresco, USA), 4-(2-hydroxyethyl)-1-piperazineethanesulfonic acid (HEPES) (25 mmol/L, Research Organics, USA), sodium bicarbonate (32 mmol/L, Research Organics, USA) and albumax II (0.5%, w/v, Life Technologies, USA). Cultures were contained in sealed T25 or T75 flasks (Corning, USA) at 5% hematocrit and flushed with a gas mix of 5% O_2_, 5% CO_2_, and 90% N_2_ (Coregas, Yennora, New South Wales). The *P. falciparum* lines used in this study were 3D7 [76], CS2 [77], 3Ci [78] and E8B [79]. HBEC and the parasite strains used were tested to be mycoplasma-free throughout the experimentation by the means of a PCR detection kit (Minerva BioLabs, Germany). For co-culture experiments, schizonts and mature trophozoites were enriched via magnetic separation [80] with an AutoMACS® (Miltenyi Biotec, Germany). The studies were performed with these late stages of the cycle because they are the predominant forms that sequester in the brain microcirculation in CM [81]

### Human brain endothelial cell culture

The human brain endothelial cell line 5i (HBEC) [16] was cultured in 25 cm^2^ (T25) or 75 cm^2^ (T75) flasks (NUNC, Denmark, pre-coated overnight with 0.1% gelatine (w/v in distilled water, Sigma-Aldrich, USA) using Dulbecco's Modified Eagle Media: Nutrient Mixture F-12 (Thermo Fisher Scientific, USA) enriched with 10% (v/v) FBS and 30 µg/mL Gentamycin (Life Technologies, USA) at 37°C with 5% CO_2_ in a humidified atmosphere.

### Peripheral blood mononuclear cell preparation

PBMCs were isolated by density gradient centrifugation from buffy coats from naïve donors obtained from the Australian Red Cross Blood Service. The buffy coat layer was first overlaid carefully onto 15 mL of Ficoll Paque Plus (GE Healthcare, USA) and centrifuged at 400 G with the brake off at room temperature for 20 min. The PBMC band was then removed and washed 3 times with RPMI1640 at 450 G for 10 min at room temperature. After washing, PBMCs were mixed with freezing medium comprised of RPMI 1640, 20% (v/v) FBS and 10% (v/v) dimethylsulfoxide (DMSO), before the freezing of aliquots in liquid nitrogen.

### Co-culture conditions

Co-culture experiments that incorporated *P. falciparum*-infected red blood cells (iRBC), peripheral blood mononuclear cells (PBMCs) and human brain endothelial cells 5i (HBEC) were performed in flat bottom microplates (Nunc, Denmark) under normoxic conditions. HBEC were seeded at 2×10^4^ per well into a 0.1% (w/v) gelatin pre-coated microplate and left to achieve confluence. The ratios of the respective cell types were chosen to be 2∶5∶100 for HBEC: PBMC: iRBC.[20] [82]. Different cell types were added simultaneously. At the end of the 24 h incubation, the co-culture experiment was terminated by centrifuging the plate at room temperature, 300 G for 3 min. The supernatant was removed for analysis of soluble products, while cells were subjected to RNA isolation by magnetic beads, followed by reverse transcription and real time PCR as described below.

### RNA isolation, reverse transcription and real time PCR

RNA was extracted using the MagMAX 96 Blood RNA isolation Kit (Life Technologies, USA) in accordance with the manufacturer's guidelines. Isolated RNA was primed with random hexamers (GeneWorks, Australia) and reverse transcribed to cDNA using Bioscript™, (Bioline, UK). Real-time PCR analyses were carried out using the Corbett Rotor-Gene 3000 (Qiagen, Germany) in KAPA SYBR® FAST Universal 1×strength qPCR Master Mix (KapaBiosystems, USA) and 100 nmol/L of each primer (see Table 1 for details). Thermal cycling conditions for the real-time PCR were: 95°C for 2 min, followed by 45 cycles of 95°C for 15 sec and 60°C for 45 sec. The purity of the PCR products was assessed by melting curve analysis and the expression of the target genes was measured using a standard curve.

**Table 1:** Primer sequences used.

	Forward	Reverse
IFN-γ	TGACCAGAGCATCCAAAAGA	TTTCGCTTCCCTGTTTTAGC
IL-10	AGAACAGCTGCACCCACT TC	GCATCACCTCCTCCAGGTAA
CXCL10	AACTGTACGCTGTACCTGCATCAGC	ACACGTGGACAAAATTGGCTTGCAG
ICAM-1	TTCACAATGACACTCAGCGGTC	AGTGCAAGCTCCCAGTGAAATG

### Cytometric bead array

IFN-γ, IL-10, CXCL10 and TNF were measured using human cytometric bead array flex sets (BD Biosciences, USA) according to the manufacturer's instructions, with the exception that all sample/reagent volumes were decreased 5-fold. Samples were run on an FC500 (Beckman Coulter, USA), and data were analysed using FlowJo (Treestar, OR) and Prism (Graphpad, CA) software. Limits of detection for the cytometric bead array are 3.7 pg/mL for IFN-γ and 0.5 pg/mL for CXCL10, as stated in the manufacturer's instructions.

### Interventions using neutralisation antibodies and inhibitors

Neutralising antibodies for IFN-γ (Clone 25718, R&D systems, USA), MHCII (Clone TU36, Becton Dickinson, USA), ICOSL (Clone 136726, R&D systems, USA) and IL-2 (Clone 5334, R&D systems, USA), as well as Orencia (Bristol-Myers, Italy), were used at 5 µg/mL, 10 µg/mL, 25 µg/mL, 5 µg/mL and 25 µg/mL respectively to block IFNγ bioactivity and/or expression. To ensure that the effects seen with the neutralising antibodies and fusion protein were specific, isotype-matched controls, IgG2A (Clone 20102), IgG1 (Clone 11711), IgG2B (Clone 20116, R&D systems, USA) and hIgG (Intragram® P, Australian Red Cross Blood Service) were used. The antibodies were pre-incubated with endothelial cells for 15 min before adding the PBMCs. After incubating PBMCs and endothelial cells together with antibody for another 30 min, iRBCs were added into the tripartite cultures for the 24 h incubation period. YVAD (Cayman Chemical, USA), also known as Z-YVAD-FMK (fluoromethylketone), is a caspase-1 inhibitor. Inclusion of YVAD at 50 µmol/L in the tripartite cultures was similar to the neutralisation antibodies, with a vehicle control of DMSO (0.135%, v/v) being included in the experimental design.

### Transwell separations

Transwell inserts of pore size 0.4 µm (Corning, USA) were used in concert with 24 well plates (Corning, USA). To optimise the growth rate of the HBEC on this artificial support, the number of cells seeded was 3.5 times more than that in a typical tripartite culture in a 96 well plate. Samples were collected after 24 h of culture, with mRNA being isolated from both the upper and lower chambers when cells were present. Concurrently, supernatants were sampled and kept at −80°C for further analysis.

### NK cell depletion

PBMCs were sorted based on their surface marker staining. Briefly, PBMCs were stained with anti-CD16 (clone 3G8, Biolegend, USA), NKp46 (clone 9E2, BioLegend, USA) and CD56 antibodies (clone MEM188, eBioscience, USA). NK cells (CD16^+^, CD56^+^, NKp46^+^) were depleted using a FACSaria cell sorter (Becton Dickinson, USA). NK-depleted PBMC were shown to be >99% NK free before inclusion in tripartite cultures.
